# Population genetic estimation of the loss of genetic diversity during horizontal transmission of HIV-1

**DOI:** 10.1186/1471-2148-6-28

**Published:** 2006-03-23

**Authors:** Charles TT Edwards, Edward C Holmes, Daniel J Wilson, Raphael P Viscidi, Elaine J Abrams, Rodney E Phillips, Alexei J Drummond

**Affiliations:** 1 Nuffield Department of Clinical Medicine, University of Oxford, The Peter Medawar Building for Pathogen Research, South Parks Road, Oxford, OX1 3SY, UK; 2 Department of Biology, The Pennsylvania State University, University Park, PA 16802, USA; 3 Department of Statistics, University of Oxford, The Peter Medawar Building for Pathogen Research, South Parks Road, Oxford, OX1 3SY, UK; 4 Department of Pediatrics, The Johns Hopkins Hospital, Baltimore, MD 21287, USA; 5 Department of Pediatrics, Columbia University College of Physicians and Surgeons and Harlem Hospital Center, NY, USA; 6 Department of Zoology, University of Oxford, South Parks Road, Oxford, OX1 3PS, UK; 7 Department of Computer Science, University of Auckland, Private Bag 92019, New Zealand

## Abstract

**Background:**

Genetic diversity of the human immunodeficiency virus type 1 (HIV-1) population within an individual is lost during transmission to a new host. The demography of transmission is an important determinant of evolutionary dynamics, particularly the relative impact of natural selection and genetic drift immediately following HIV-1 infection. Despite this, the magnitude of this population bottleneck is unclear.

**Results:**

We use coalescent methods to quantify the bottleneck in a single case of homosexual transmission and find that over 99% of the *env *and *gag *diversity present in the donor is lost. This was consistent with the diversity present at seroconversion in nine other horizontally infected individuals. Furthermore, we estimated viral diversity at birth in 27 infants infected through vertical transmission and found there to be no difference between the two modes of transmission.

**Conclusion:**

Assuming the bottleneck at transmission is selectively neutral, such a severe reduction in genetic diversity has important implications for adaptation in HIV-1, since beneficial mutations have a reduced chance of transmission.

## Background

The size of the inoculum that initiates infection in HIV-1 is unknown, although the loss of diversity is thought to be substantial following both horizontal [1-7] and vertical [8,9] transmission. If the bottleneck is selectively neutral, genetic drift will occur because only a small number of variants are chosen at random from the population to propagate the new infection. The smaller the amount of genetic diversity transmitted the greater the magnitude of drift, lowering the probability that adaptive changes that emerge within hosts will survive transmission.

In RNA viruses with a high deleterious mutation rate the majority of variants exhibit a replicative capacity lower than the mean [10-12]. Because the fittest variants may only be present at a low frequency, they are susceptible to random loss. Hence when genetic drift is strong, deleterious mutations may accumulate, leading to an irreversible decline in population fitness [13]. Although the high rate of recombination in HIV-1 *in vivo *[14-16] has the potential to rescue debilitated haplotypes [13], if a new infection is initiated by only one or a few viral particles, and if these are chosen at random from the parent population, then the transmission of HIV-1 will likely incur a substantial reduction in fitness [17-21]. As the inoculum size increases, potential fitness losses are rapidly reduced [22,23].

Conversely, natural selection may lower the susceptibility of HIV-1 to reductions of fitness associated with transmission. In acutely infected HIV-1 patients, the usually diverse envelope *V3 *region is more homogeneous than *gag p17*, whereas in chronic infection the opposite is true [6,7]. Positive selection operating on envelope during transmission has been invoked as an explanation [6,7]. If selection operates to influence which variants are transmitted then it will also prevent the fixation of deleterious mutations.

Herein we estimate, using population genetic techniques, the proportion of genetic diversity that survives transmission in a single homosexual transmitter pair, with samples available before and after the transmission event. The demographic history of the virus population in both donor and recipient was reconstructed using coalescent methodology, allowing quantification of the diversity present close to the time of infection. The coalescent was implemented within a Bayesian framework, which enabled co-estimation of substitution and demographic parameters using serially sampled sequences [24-26].

Through a comparison of different regions of the genome (namely *env V1-V4 *and *gag p24*) we also investigate whether selection is likely to be acting during HIV-1 transmission. Finally, we generalise our result by estimating the diversity present close to the time of infection in nine homosexual seroconverters for which donor sequences were unavailable, and compare horizontal and vertical modes of transmission using 27 infants infected at birth.

## Results

**Figure 1:**
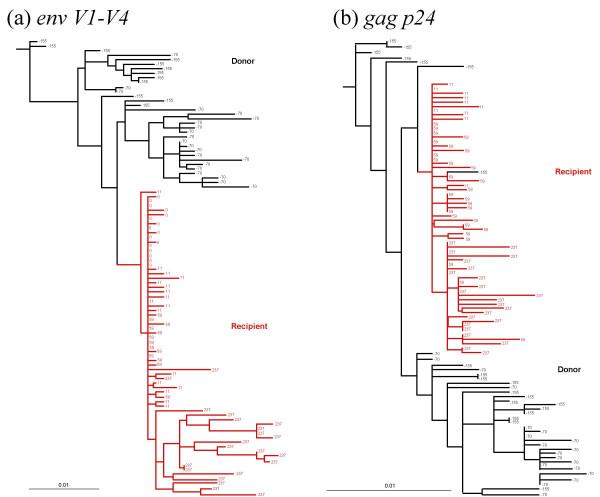
**Phylogenetic relationship of (a) *env V1-V4 *and (b) *gag p24 *sequences**. Maximum likelihood phylogenies depicting the relationship between sequences from donor and recipient, illustrating the reduction in genetic diversity at transmission. Horizontal branch lengths are drawn on a scale of nucleotide changes per site. Branches leading to recipient sequences are highlighted in red, with the day of sample collection relative to the first recipient sample (day 0) shown for each sequence.

To directly visualise the change in genetic diversity during horizontal HIV-1 transmission between the donor-recipient pair studied, we first inferred the phylogenetic relationships among their HIV-1 sequences using maximum likelihood methods. The phylogenies for *env V1-V4 *and *gag p24 *depicted in Figure 1 show that branch lengths are substantially shortened immediately after transmission, illustrating that a significant reduction in diversity has occurred.

To investigate the demographics of viral transmission in this transmitter pair more closely, four coalescent models were fitted to the sequence data. Crucially, samples were available both before and after the transmission event allowing distinct demographic functions for donor and recipient HIV-1 populations (Equations 1 to 5), with the time of transition between them estimated from the data [26]. In addition to a null model that constrained the effective population size in donor (*N*_*D*_) and recipient (*N*_*R*_) to be identical (so that there is no bottleneck at transmission), models with constant, exponential and logistic demographic functions for the recipient population were fitted. In all cases the donor population size was assumed to be constant.

**Figure 2:**
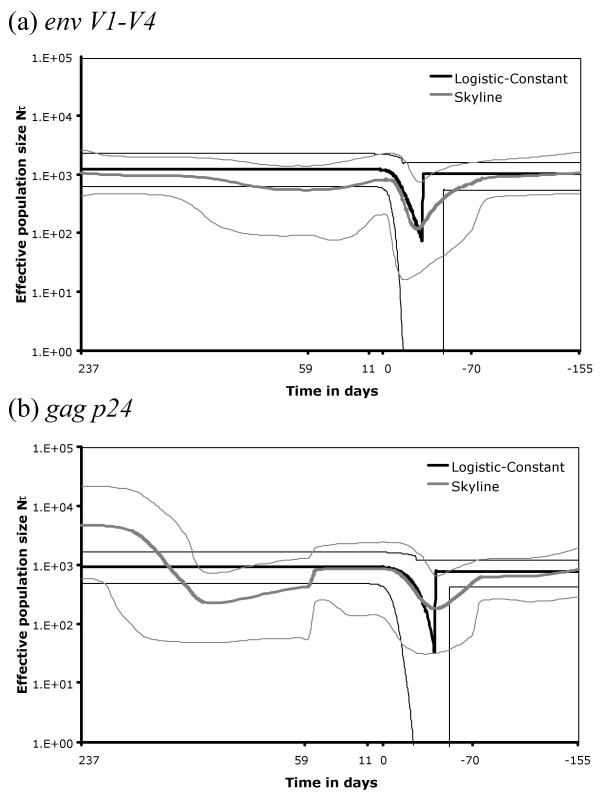
**Reconstructed demographic profiles for (a) *env V1-V4 *and (b) *gag p24***. Estimates of *Nτ *are shown on a log scale against time backwards since the most recent sample. Only days on which sequences were sampled are shown, measured relative to the first recipient sample (day 0). Mean estimates of *Nτ *obtained from the best fit Logistic-Constant demographic model and the Bayesian skyline plot are shown with their HPD confidence bounds.

The relative Bayesian posterior scores for each demographic model are listed in Table 1. For both *env V1-V4 *and *gag p24*, the model with the lowest AIC (the preferred model) fits a constant population size in the donor and logistic growth in the recipient (Equations 4 and 5). The null hypothesis that there has been no change in population size at transmission was therefore rejected. Using the estimated model parameters we reconstructed the demographic profiles of genetic diversity (*Nτ*, the product of the effective population size and generation length in days [27]) against time for each gene (Figure 2).

**Table 1:** Fit of demographic models

Demographic Model				Coalescent
Recipient	Donor	*lnLk*^b^	AIC^c^	ESS^d^
*env V1-V4*				
			
**Constant**	**-**^a^	-4155.385	8310.77	523.85
**Constant**	**Constant**	-4144.993	8291.99	419.77
**Exponential**	**Constant**	-4103.717	8211.43	643.10
**Logistic**	**Constant**	-4090.154	8186.31	126.43
				
*gag p24*				
			
**Constant**	**-**^a^	-3118.180	6236.36	483.84
**Constant**	**Constant**	-3121.900	6245.80	440.34
**Exponential**	**Constant**	-3116.760	6237.52	378.33
**Logistic**	**Constant**	-3089.852	6185.70	202.69

^a^Population size in Recipient constrained to be the same as that in Donor

^b^Natural logarithm of the likelihood obtained from fitting the demographic model to the data

^c^Akaike Information Criteria

^d^Effective Sample Size (number of independent coalescent genealogies sampled from the posterior distribution)

To further test the extent of the transmission bottleneck, the demographic history of the population was reconstructed using the Bayesian skyline plot [see Methods, [28]]. The results for *env V1-V4 *and *gag p24 *are shown in Figure 2. In both cases there is a good fit between the demographic profiles estimated using the two different methods. Noticeably, the timing of the transmission bottleneck is the same, and evidence for a bottleneck is readily apparent under both models.

**Figure 3:**
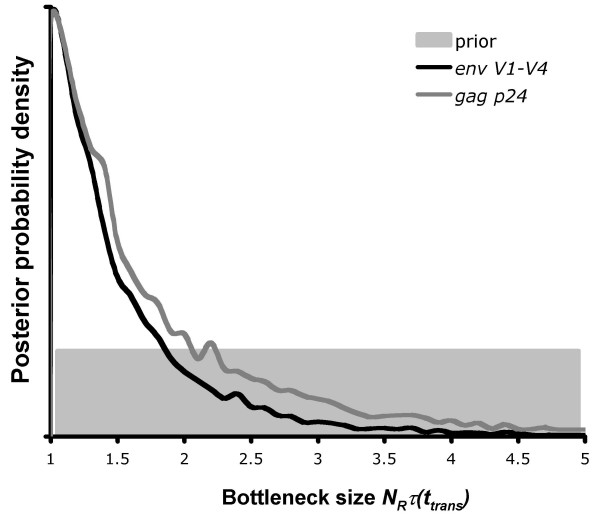
**Effective population size at transmission *N*_*R*_*τ*(*t*_*trans*_)**. The marginal posterior probability density of *N*_*R*_*τ*(*t*_*trans*_) is shown for both *env V1-V4 *and *gag p24*. The shaded area represents the uniform prior distribution that was used, with a minimum bound of one.

The Bayesian skyline plot also justifies our use of the logistic-constant demographic model to estimate the diversity that survives during horizontal transmission of HIV-1. Using the logistic growth model (Equations 4 and 5) we were able to calculate diversity in the recipient *N*_*R*_*τ *at the estimated time of transmission *t*_*trans*_. We estimated *t*_*trans *_to be approximately 30 days prior to collection of the first recipient sample (day 0) for *env *and 40 days for *gag *(Table 2). We calculated *N*_*R*_*τ*(*t*_*trans*_) to be 1.6 for *env V1-V4*, and 2.0 for *gag p24 *(Table 2). These values are near the lower prior boundary of one and their posterior distributions both exhibit a large positive skew (Figure 3). The level of diversity in the donor at the time of transmission *N*_*D*_*τ *was compared with that which was transmitted *N*_*R*_*τ*(*t*_*trans*_) as a percentage ratio *δ*. For *env*, *N*_*D*_*τ *was estimated to be 1014, giving a value of *δ *as 0.17%. For *gag p24*, *N*_*D*_*τ *was 771, giving *δ *as 0.29% (Table 2).

**Table 2:** Parameter estimates used to calculate the percentage diversity that survived transmission

Parameter	Mean^a^	HPD^b ^Lower	HPD Upper	ESS^c^
*env V1-V4*				
				
*N*_*R*_*τ*	1216.7	534.4	2033.9	1338.94
*N*_*D*_*τ*	1014.0	541.2	1538.0	955.71
*t*_*trans*_^d^	30.9	15.2	46.9	174.57
*N*_*R*_*τ*(*t*_*trans*_)	1.6	1.0	3.1	2456.87
***δ ***	0.17	0.06	0.35	2043.08
				
*gag p24*				
				
*N*_*R*_*τ*	926.6	419.9	1512.6	1454.31
*N*_*D*_*τ*	770.7	413.5	1184.7	1356.90
*t*_*trans*_^d^	42.4	27.5	53.0	274.51
*N*_*R*_*τ*(*t*_*trans*_)	2.0	1.0	4.5	3532.69
***δ ***	0.29	0.07	0.67	3310.56

^a^Mean of the marginal posterior probability distribution of parameter values

^b^Highest Posterior Density encompassing 95% of the marginal posterior distribution of parameter values

^c^Effective Sample Size (number of independent samples taken from the posterior distribution of values for a particular parameter)

^d^Estimated time of transmission in days prior to the day of the fist recipient sample (day 0)

Importantly, if selection was acting on *env *to restrict the proportion of variants capable of establishing a new infection, we would expect a greater loss of diversity in this region when compared to *gag*, assuming recombination between the two regions. Therefore, the similarity in *δ *between *env *and *gag *argues against strong selection at transmission.

We conclude that > 99% of genetic diversity in the donor viral population, in both *env *and *gag*, was lost during this case of horizontal transmission. A reduction in viral diversity after horizontal transmission has been reported frequently in the literature [1,3-6]. However, information regarding the diversity present in the donor is often lacking, and even in cases where this data exists [2,7] it is difficult to measure levels of diversity close to the transmission event. The method implemented here overcomes this problem, estimating genetic diversity at the inferred time of transmission, and therefore allows accurate quantification of the transmission bottleneck.

To generalise this result we next investigated diversity (*Nτ*) of the founding viral population in nine patients infected through homosexual contact for which donor sequences were unavailable. Sequences had been published previously [29]. Assuming the best-fit demographic model, *Nτ *at seroconversion was found to vary between around 1720 and 8 (mean: 406; Table 3). In the recipient of the transmitter pair, *Nτ *at seroconversion (day 0) was 1150 (HPD upper: 1930), which is not significantly different (*p *= 0.302; one-sample *t*-test).

**Table 3:** Estimates of viral diversity close to the time of transmission

Patient	Best-fitting demographic model	*μ*^a^	*Nτ *^b^	*Nτ *close to transmission^c^	
				
				Mean^d^	HPD^e ^upper
Horizontal transmission					
				
p1	Logistic	0.0123	2293	36.00	153.19
p2	Logistic	0.0166	4441	27.98	148.32
p3	Logistic	0.0175	1612	29.02	80.12
p5	Exponential	0.0223	2439	287.78	670.67
p6	Logistic	0.0195	1511	7.86	20.39
p7	Logistic	0.0085	8632	253.78	1173.95
p8	Exponential	0.0162	6003	1722.98	2911.65
p9	Logistic	0.0071	7168	1283.11	3211.35
p11	Logistic	0.0128	6505	9.76	34.48
**Mean**		0.0148	4512	406.47	933.79
					
Vertical transmission					
				
p1	Logistic	0.0201	4183	15.48	79.99
p2	Constant	0.0560	275	275.46	511.97
p3	Constant	0.0163	383	383.27	714.69
p4	Exponential	0.0098	67696	1214.76	5810.63
p5	Exponential	0.0133	7372	1360.46	3444.98
p6	Constant	0.0251	183	181.98	323.10
p7	Exponential	0.0226	1165	151.26	294.62
p8	Constant	0.0145	521	521.84	857.01
p9	Exponential	0.0120	2740	191.34	405.22
p10	Logistic	0.0188	1050	384.47	877.21
p11	Logistic	0.0163	740	410.67	926.82
p12	Exponential	0.0206	1730	121.83	254.40
p13	Exponential	0.0218	2603	81.27	173.89
p14	Exponential	0.0164	1865	208.00	411.70
p15	Logistic	0.0397	889	1.55	4.80
p16	Logistic	0.0173	269904	261.90	577.67
p18	Logistic	0.0097	146723	960.34	1978.63
p19	Exponential	0.0095	2842	342.42	655.38
p21	Exponential	0.0053	302840	1712.90	4936.78
p22	Exponential	0.0046	547670	3159.60	11050.00
p23	Logistic	0.0093	123360	371.85	706.09
p24	Logistic	0.0102	97018	524.69	814.59
p25	Logistic	0.0071	2508	2194.63	4321.04
pa	Constant	0.0280	640	638.53	895.76
pb	Constant	0.0076	2006	2000.48	3593.83
pc	Constant	0.0094	879	879.45	1626.67
pd	Constant	0.0146	254	254.26	526.95
**Mean**		0.0169	58890	696.47	1732.39

^a^Substitution rate in number of changes per site per year

^b^Product of the effective population size and generation time in days at the most recent time point

^c^Seroconversion or birth for horizontally and vertically infected patients respectively

^d^Mean of the marginal posterior probability distribution of parameter values

^e^Highest Posterior Density

Finally, to compare the diversity present close to the time of infection in patients infected via two different modes of transmission, we estimated *Nτ *at birth (transmission) in 27 vertically infected infants. The average *Nτ *at birth was 696 (Table 3). Although we were unable to detect a bottleneck at transmission in eight of the infants (p2, p3, p6, p8, pa, pd, pc and pd), the estimates for *Nτ *close to the time of infection in the horizontally and vertically infected patient groups were not significantly different (*p *= 0.320; two-sample *t*-test).

## Discussion

From our analysis of a single donor and recipient transmission pair, we conclude that in this case the viral diversity sampled during homosexual transmission of HIV-1 was very small (< 1%). This result was consistent for both *env *and *gag*. Interpretation of our finding is dependent on whether transmission is considered a neutral or selective process. In particular, if transmission is neutral it can be concluded from the severe bottleneck reported here that the consequent genetic drift will be strong, with negative consequences for viral fitness. Natural selection on the other hand is likely to mitigate any deleterious effects of genetic drift associated with transmission.

It is possible that the diversity present in the inoculum itself was larger, and that selection acting on *env *restricted propagation of the new infection to a few members of the initial population [30]. The similar levels of diversity observed in *env *and *gag *could then be explained by genetic coupling between the two regions. The frequency with which recombination occurs in HIV-1 [14-16] argues against such linkage, suggesting that independent selective forces acting on *env *and *gag *must be invoked to explain this observation. Alternatively, if transmission is neutral then our estimate of the diversity transmitted will be closer to the diversity actually present in the inoculum. This will have implications for the replicative fitness of the viral population responsible for founding a new infection. Indeed, it has been shown experimentally that a random population bottleneck of a single clone can have severe consequences for the replicative fitness of HIV-1 [21]. Furthermore, by lowering their chances of transmission, genetic drift has the potential to prevent the accumulation of advantageous changes at the population level, thereby impeding the long-term adaptation of HIV-1 [31].

Neutral transmission also means that the degree of genetic diversity passed between individuals is dependent on the diversity present in the donor at the time of transmission. Because diversity in their respective donors is likely to vary greatly depending on the stage of infection [29], this could in part explain our finding of wide variation across patients in diversity of the viral population close to transmission (Table 3). Furthermore, we found the degree of variability across patients infected by the same route to be greater than any difference between groups infected via different modes of transmission (i.e. the difference between groups was not significant). Interestingly, the diversity present early in acute infection in sexually and parenterally infected individuals also appears similar [2].

We can conclude from our results that diversity of the founding population is similarly restricted during both horizontal and vertical transmission. However, it is also clear that further study is required to investigate the variability observed. For example, although a reduction in diversity is frequent [1-9], the transmission of multiple variants has also been reported during both horizontal [32] and vertical transmission [33-35], suggesting that the bottleneck is not universally restrictive.

## Conclusion

Our findings quantify the contraction in genetic diversity that occurs during horizontal transmission of HIV-1. It is clear from the severity of the bottleneck that further work is required to investigate the nature of the selective forces surrounding transmission, if we are to interpret the fitness consequences for HIV-1 in the newly infected individual. Furthermore, the analyses presented suggest that the mode of transmission may not be a significant influence on the genetic diversity transmitted.

## Methods

### Patient material

The donor and recipient patients of the transmitter pair analysed here were recruited as part of an on-going study of acute HIV-1 infection and have been described in detail elsewhere [36]. The donor had been infected for at least two years prior to transmission and exhibited a stable viral load. He had not received any antiretroviral treatment. The recipient was also untreated during the time of sampling but progressed rapidly towards disease with high viral loads and low CD4^+ ^cell counts. The clinical data for both donor and recipient during sampling is given in Additional file 1.

The first recipient sample (day 0) was collected six weeks after he last tested PCR (polymerase chain reaction) negative for HIV-1 DNA and RNA. Three additional samples from the recipient were available at days 11, 59 and 237. Donor samples were collected 70 and 155 days prior to the first sample from the recipient. *Gag p24 *(834bp) and the *V1-V4 *region of the *env *gene (951bp) were sequenced from viral RNA. Envelope sequences were obtained from all time points using previously described methods [37], yielding a total of 100 clones (average: 17 clones per time point; range: 12–21). For *gag p24*, a total of 100 clones were sequenced from all time points except day 0 (average: 20 clones per time point; range: 10–28). Details of viral loads from each time point are listed in Additional file 1. Sequences are available from GenBank under accession numbers DQ316399–DQ316601.

Envelope sequences were also obtained from 27 HIV-1 positive children. All were HIV negative by PCR at birth indicating that infection occurred *peri-partum *rather than *in utero*. Detailed descriptions of the cohort [38,39] and sequencing techniques [40] are given elsewhere. The clinical prognosis of each patient is given in Additional file 2. Sequences were around 360bp in length, spanning the highly variable envelope V3 region. Multiple clones were collected from serial time points post-infection (Additional file 2). All sequences (excepting those from pa, pb, pc and pd) were derived from viral RNA. These sequences are available from GenBank under accession numbers AY823998–AY824946.

### Phylogenetic inference

Sequences were first aligned manually using Se-Al [41]. Maximum likelihood phylogenies for *env V1-V4 *and *gag p24 *sequences were then constructed using PAUP* [42]. Estimation assumed the HKY85 + I + dΓ_4 _model of nucleotide substitution [43,44]. All parameters were inferred from the data using maximum likelihood.

### Quantification of the diversity lost during horizontal transmission

Within a coalescent framework, and assuming the HKY85 + dΓ_4 _model of nucleotide substitution [43,44], four demographic models were fitted to the transmission pair sequence data.

Null model: *N*_*t *_= *N*_*R *_= *N*_*D *_    [1]

Constant-Constant:Nt={NRt≤ttransNDt>ttrans     [2]
 MathType@MTEF@5@5@+=feaafiart1ev1aaatCvAUfKttLearuWrP9MDH5MBPbIqV92AaeXatLxBI9gBaebbnrfifHhDYfgasaacH8akY=wiFfYdH8Gipec8Eeeu0xXdbba9frFj0=OqFfea0dXdd9vqai=hGuQ8kuc9pgc9s8qqaq=dirpe0xb9q8qiLsFr0=vr0=vr0dc8meaabaqaciaacaGaaeqabaqabeGadaaakeaafaqabeqacaaabaGaee4qamKaee4Ba8MaeeOBa4Maee4CamNaeeiDaqNaeeyyaeMaeeOBa4MaeeiDaqNaeeyla0Iaee4qamKaee4Ba8MaeeOBa4Maee4CamNaeeiDaqNaeeyyaeMaeeOBa4MaeeiDaqNaeiOoaOdabaGaemOta40aaSbaaSqaaiabdsha0bqabaGccqGH9aqpdaGabaqaauaabeqaciaaaeaacqWGobGtdaWgaaWcbaGaemOuaifabeaaaOqaaiabdsha0jabgsMiJkabdsha0naaBaaaleaacqWG0baDcqWGYbGCcqWGHbqycqWGUbGBcqWGZbWCaeqaaaGcbaGaemOta40aaSbaaSqaaiabdseaebqabaaakeaacqWG0baDcqGH+aGpcqWG0baDdaWgaaWcbaGaemiDaqNaemOCaiNaemyyaeMaemOBa4Maem4CamhabeaaaaaakiaawUhaaaaacaWLjaGaaCzcaiabcUfaBjabikdaYiabc2faDbaa@69BB@

Exponential-Constant:Nt={NRe−rtt≤ttransNDt>ttrans     [3]
 MathType@MTEF@5@5@+=feaafiart1ev1aaatCvAUfKttLearuWrP9MDH5MBPbIqV92AaeXatLxBI9gBaebbnrfifHhDYfgasaacH8akY=wiFfYdH8Gipec8Eeeu0xXdbba9frFj0=OqFfea0dXdd9vqai=hGuQ8kuc9pgc9s8qqaq=dirpe0xb9q8qiLsFr0=vr0=vr0dc8meaabaqaciaacaGaaeqabaqabeGadaaakeaacqqGfbqrcqqG4baEcqqGWbaCcqqGVbWBcqqGUbGBcqqGLbqzcqqGUbGBcqqG0baDcqqGPbqAcqqGHbqycqqGSbaBfaqabeqacaaabaGaeeyla0Iaee4qamKaee4Ba8MaeeOBa4Maee4CamNaeeiDaqNaeeyyaeMaeeOBa4MaeeiDaqNaeiOoaOdabaGaemOta40aaSbaaSqaaiabdsha0bqabaGccqGH9aqpdaGabaqaauaabeqaciaaaeaacqWGobGtdaWgaaWcbaGaemOuaifabeaakiabdwgaLnaaCaaaleqabaGaeyOeI0IaemOCaiNaemiDaqhaaaGcbaGaemiDaqNaeyizImQaemiDaq3aaSbaaSqaaiabdsha0jabdkhaYjabdggaHjabd6gaUjabdohaZbqabaaakeaacqWGobGtdaWgaaWcbaGaemiraqeabeaaaOqaaiabdsha0jabg6da+iabdsha0naaBaaaleaacqWG0baDcqWGYbGCcqWGHbqycqWGUbGBcqWGZbWCaeqaaaaaaOGaay5EaaaaaiaaxMaacaWLjaGaei4waSLaeG4mamJaeiyxa0faaa@7321@

Logistic-Constant:Nt={NR(1+c)e−rtc+e−rtt≤ttransNDt>ttrans     [4]
 MathType@MTEF@5@5@+=feaafiart1ev1aaatCvAUfKttLearuWrP9MDH5MBPbIqV92AaeXatLxBI9gBaebbnrfifHhDYfgasaacH8akY=wiFfYdH8Gipec8Eeeu0xXdbba9frFj0=OqFfea0dXdd9vqai=hGuQ8kuc9pgc9s8qqaq=dirpe0xb9q8qiLsFr0=vr0=vr0dc8meaabaqaciaacaGaaeqabaqabeGadaaakeaacqqGmbatcqqGVbWBcqqGNbWzcqqGPbqAcqqGZbWCcqqG0baDcqqGPbqAcqqGJbWyfaqabeqacaaabaGaeeyla0Iaee4qamKaee4Ba8MaeeOBa4Maee4CamNaeeiDaqNaeeyyaeMaeeOBa4MaeeiDaqNaeiOoaOdabaGaemOta40aaSbaaSqaaiabdsha0bqabaGccqGH9aqpdaGabaqaauaabeqaciaaaeaadaWcaaqaaiabd6eaonaaBaaaleaacqWGsbGuaeqaaOGaeiikaGIaeGymaeJaey4kaSIaem4yamMaeiykaKIaemyzau2aaWbaaSqabeaacqGHsislcqWGYbGCcqWG0baDaaaakeaacqWGJbWycqGHRaWkcqWGLbqzdaahaaWcbeqaaiabgkHiTiabdkhaYjabdsha0baaaaaakeaacqWG0baDcqGHKjYOcqWG0baDdaWgaaWcbaGaemiDaqNaemOCaiNaemyyaeMaemOBa4Maem4CamhabeaaaOqaaiabd6eaonaaBaaaleaacqWGebaraeqaaaGcbaGaemiDaqNaeyOpa4JaemiDaq3aaSbaaSqaaiabdsha0jabdkhaYjabdggaHjabd6gaUjabdohaZbqabaaaaaGccaGL7baaaaGaaCzcaiaaxMaacqGGBbWwcqaI0aancqGGDbqxaaa@7B65@

where c=1ert50−2     [5]
 MathType@MTEF@5@5@+=feaafiart1ev1aaatCvAUfKttLearuWrP9MDH5MBPbIqV92AaeXatLxBI9gBaebbnrfifHhDYfgasaacH8akY=wiFfYdH8Gipec8Eeeu0xXdbba9frFj0=OqFfea0dXdd9vqai=hGuQ8kuc9pgc9s8qqaq=dirpe0xb9q8qiLsFr0=vr0=vr0dc8meaabaqaciaacaGaaeqabaqabeGadaaakeaacqqG3bWDcqqGObaAcqqGLbqzcqqGYbGCcqqGLbqzcqqGGaaicqWGJbWycqGH9aqpdaWcaaqaaiabigdaXaqaaiabdwgaLnaaCaaaleqabaGaemOCaiNaemiDaq3aaSbaaWqaaiabiwda1iabicdaWaqabaaaaOGaeyOeI0IaeGOmaidaaiaaxMaacaWLjaGaei4waSLaeGynauJaeiyxa0faaa@44B7@

All substitution and demographic parameters, including the time of transmission *t*_*trans*_, growth rate *r*, and mid-time of the population *t*_50_, were estimated from the data within a Bayesian coalescent framework by Markov chain Monte Carlo (MCMC), using the BEAST program [45]. Bayesian MCMC estimates each parameter as the mean of its marginal posterior probability distribution, whilst simultaneously incorporating uncertainty in the underlying genealogy and other parameters. Diversity of the viral population is given as the product of the effective population size and generation length in days *Nτ *[27].

Uncertainty in the estimated parameter values is summarized by the highest posterior density (HPD) interval, which contains 95% of the marginal posterior distribution. The length of the MCMC chain was chosen so that the effective sample size (ESS) for each parameter was > 100, indicating that parameter space had been sufficiently explored [24]. Since it consistently gave the lowest value, the coalescent ESS (the number of effectively independent log likelihoods sampled from the coalescent posterior distribution) for each model is given in Table 1. All priors were assumed to be uniform on a natural scale, including the effective population size in the recipient at transmission *N*_*R*_*τ*(*t*_*trans*_). The prior boundaries for the time of transmission *t*_*trans *_were set from when the recipient was last confirmed HIV-1 negative (53 days before the first recipient sample) to the time at which the first recipient sample was collected (day 0). We placed a minimum prior bound of one on *N*_*R*_*τ*(*t*_*trans*_). With the exception of *t*_*trans *_and *N*_*R*_*τ*(*t*_*trans*_), the MCMC chain did not impinge on any of the prescribed prior boundaries for the models tested.

The relative fit of each model to the data was assessed using the Akaike Information Criteria (AIC) [46]. The AIC of a given model is twice its marginal log likelihood plus the number of parameters specified (AIC = 2*lnLk *+ 2*p*). The model with the lowest AIC is selected as the best representation of the data.

Selection of the appropriate demographic model allowed us to calculate *N*_*R*_*τ*(*t*_*trans *_) and quantify the amount of diversity lost at transmission through a comparison of *N*_*R*_*τ*(*t*_*trans *_) with *N*_*D*_*τ *as the percentage ratio *δ*.

### Bayesian skyline plot

The skyline plot is a piecewise-constant model of population size that estimates *Nτ *for each coalescent interval of the genealogy [47,48]. It allows the demographic history of a population to be reconstructed without *a priori *specification of a particular model. The Bayesian skyline extends the generalised skyline plot [48] to take into account serial sequence sampling times and an uncertain genealogy [28]. The distribution of skyline plots is sampled using MCMC according to their posterior probabilities given the sequence data, to produce an estimate and HPD confidence intervals of the effective population size through time. The Bayesian skyline plot was estimated using BEAST [45], allowing ten steps in *Nτ *through time.

## Authors' contributions

CTTE collected the data, performed the analysis and wrote the paper. ECH contributed to the design of the study and the writing of the manuscript. DJW assisted with the analysis. RPV and EJA collected sequences for the infant data set. REP contributed to the design of the study and provided the funding. AJD contributed to the design of the study, the development of the software, the analysis and the writing of the article.

## Supplementary Material

Additional File 1 Clinical data for transmission pair Click here for file

Additional File 2 Clinical categorisation and sequencing profile of vertically infected infants Click here for file
