# Supplementary material for: Population genetic estimation of the loss of genetic diversity during horizontal transmission of HIV-1
Source: BMC Evol Biol. 2006 Mar 23;6:28. doi: 10.1186/1471-2148-6-28 (PMC1444934; doi:10.1186/1471-2148-6-28)
Supplement: Additional File 1 — Clinical data for transmission pair [file 1471-2148-6-28-S1.doc]

Additional File 1 – Clinical data for transmission pair

|  | Day of sample | Viral load copies/ml | CD4^+^ cell count  cells/µl |
| --- | --- | --- | --- |
| Recipient | 237 | 1289725 | 260 |
|  | 59 | 816964 | 290 |
|  | 11 | 910600 | 350 |
|  | 0 | 15934216 | 170 |
| Donor | -70 | 25221 | 590 |
|  | -155 | 50517 | 610 |
