# Supplementary material for: Population genetic estimation of the loss of genetic diversity during horizontal transmission of HIV-1
Source: BMC Evol Biol. 2006 Mar 23;6:28. doi: 10.1186/1471-2148-6-28 (PMC1444934; doi:10.1186/1471-2148-6-28)
Supplement: Additional File 2 — Clinical categorisation and sequencing profile of vertically infected infants [file 1471-2148-6-28-S2.doc]

### Additional File 2 – Clinical categorisation and sequencing profile of vertically infected infants

| Patient | Time after birth of first sample in days | Total sampling time in days | No. of sequences | Clinical prognosis^a^ |
| --- | --- | --- | --- | --- |
|  |  |  |  |  |
| **p1** | 69 | 644 | 23 | MNP |
| **p2** | 47 | 378 | 21 | MP |
| **p3** | 24 | 882 | 22 | SNP |
| **p4** | 28 | 343 | 28 | MNP |
| **p5** | 71 | 668 | 35 | MNP |
| **p6** | 31 | 713 | 32 | MNP |
| **p7** | 89 | 871 | 38 | SNP |
| **p8** | 53 | 848 | 40 | MP |
| **p9** | 11 | 1050 | 28 | MNP |
| **p10** | 25 | 989 | 58 | MP |
| **p11** | 11 | 968 | 36 | SNP |
| **p12** | 24 | 1064 | 35 | SNP |
| **p13** | 20 | 944 | 44 | MP |
| **p14** | 38 | 1047 | 35 | MNP |
| **p15** | 181 | 742 | 42 | SNP |
| **p16** | 23 | 1041 | 32 | SNP |
| **p18** | 2 | 550 | 33 | RP |
| **p19** | 24 | 1052 | 41 | SNP |
| **p20** | 123 | 252 | 14 | RP |
| **p21** | 10 | 887 | 28 | RP |
| **p22** | 120 | 629 | 16 | RP |
| **p23** | 29 | 734 | 38 | RP |
| **p24** | 61 | 506 | 39 | RP |
| **p25** | 29 | 274 | 21 | MP |
| **pa** | 220 | 1668 | 70 | MP |
| **pb** | 64 | 905 | 40 | SNP |
| **pc** | 7 | 383 | 38 | SNP |
| **pd** | 33 | 342 | 22 | RP |
|  |  |  |  |  |

^a^Patients were categorised based on CD4^+^ T cell counts and a clinical diagnosis of AIDS. Rapid progressors (RP) were defined as infants who were clinically diagnosed with AIDS and had < 250 CD4^+^ T cells per ml within 18 months of birth. Infants in this category eventually succumbed to their disease (excepting p24). Infants diagnosed with AIDS but with higher CD4^+^ T cell counts (from 470 to 2338 cells at 18 months) were classified as moderate progressors (MP). One infant in this category died at 20 months (p2). The remaining infants did not develop AIDS throughout the period of study, or during a 2.5 to 8 year follow-up. Those with < 1500 CD4^+^ T cells per ml at 24 months were classified as moderate non-progressors (MNP), and with > 1500 CD4^+^ T cells per ml as slow non-progressors (SNP).
